# Barriers to the Use of the Mental Health Treatment Requirement as Part of a Community-Based Criminal Sentence for Mentally Disordered Offenders

**DOI:** 10.1192/bjo.2022.111

**Published:** 2022-06-20

**Authors:** Fatima Saleem, Hector Blott

**Affiliations:** 1 St George's University, London, United Kingdom; 2 South West London and St George's Mental Health NHS Trust, London, United Kingdom

## Abstract

**Aims:**

Due to the high rates of mental disorder in prison there have been a number of initiatives to divert mentally disordered offenders out of custody. One of these is the Mental Health Treatment Requirement (MHTR): a criminal sentence available as part of a Community Order, offered as an alternative to short-term custodial sentences in an attempt to address recidivism and encourage concordance with community psychiatric treatment. In spite of the prevalence of mental disorder amongst offenders, MHTRs represent less than 1% of all community sentences. Here we aim to identify obstacles to the use of the MHTR at sentencing and to suggest ways of overcoming them.

**Methods:**

A literature review and brief case series will be used to identify and illustrate what may be obstructing or limiting the use of the MHTR. The terms ‘MHTR’ and ‘Mental Health Treatment Requirement’ were searched on Google, Google Scholar, Athens, and PubMed, and results analysed for recurrent themes. The issues encountered clinically by one of the authors who referred three defendants for MHTRs in psychiatric sentencing reports in 2021 were reviewed with the same purpose.

**Results:**

The main barriers to the use of the MHTR which were identified were issues related to a lack of clinician awareness and experience, homelessness and housing, and service structure and provision. There may be a reluctance for Community Mental Health Teams (CMHTs) to accept offenders onto their caseloads, and there are challenges in obtaining assessments and recommendations for MHTRs. There are difficulties in securing an MHTR for homeless defendants on remand for whom identifying housing prior to sentencing, and thus a CMHT to supervise the MHTR, can be challenging. The MHTR assessment and referral process is more lengthy and cumbersome than that for most other disposals, leaving the defendant awaiting sentencing (potentially in custody) while the referral is processed.

**Conclusion:**

Suggested solutions to improve access to the MHTR include increasing clinician awareness and confidence by providing teaching and training, and multi-agency meetings to enhance communication and create an understanding amongst professionals of duties, roles, and responsibilities. Initiatives to identify housing for homeless remand prisoners before their release, as well as ensuring the availability of community services to supervise treatment, would overcome some of the obstacles identified and increase availability of the MHTR. Funding for additional staff to conduct the assessment and referrals process would also be likely to improve uptake.

